# The role of urine chloride in acute heart failure

**DOI:** 10.1038/s41598-024-64747-5

**Published:** 2024-06-18

**Authors:** Sylwia Nawrocka-Millward, Jan Biegus, Marat Fudim, Mateusz Guzik, Gracjan Iwanek, Piotr Ponikowski, Robert Zymliński

**Affiliations:** 1 University Clinical Hospital, Wroclaw, Poland; 2 Institute of Heart Diseases, Wroclaw Medical University, Wroclaw, Poland; 3 Division of Cardiology, Duke University Medical Center, Durham, NC USA; 4 Duke Clinical Research Institute, Durham, NC USA

**Keywords:** Biomarkers, Cardiology

## Abstract

In our retrospective study, we aimed to investigate the relationship between urinary chloride (uCl^−^) and selected clinical and laboratory biomarkers, renal function, and patient outcomes in the acute heart failure (AHF) population. We divided 248 adult patients (≥ 18 years) with AHF into two groups: low uCl^−^ (< 115 mmol/L) and high uCl^−^. The mean age of the patient group was 70.2 ± 12.6, and 182 patients were male (73.4%). Clinical endpoints included in-hospital mortality, one-year mortality, and a composite endpoint of one-year mortality and rehospitalization for heart failure. Patients were followed up for at least one year. Relevant clinical and baseline biomarker data were collected, including markers concerning inflammation, liver and kidney function, perfusion and congestion, iron status, cardiac remodeling, gasometry, renin and aldosterone. Low uCl^−^ was associated with worse in-hospital outcomes, including higher in-hospital mortality (7.7% vs. 1.4%, p = 0.014), the need for inotropic support (20.19% vs. 2.08%, p ≤ 0.001), worsening of HF during therapy (17.31% vs. 4.86%, p ≤ 0.001), and the need for treatment in an intensive cardiac care unit (33.65% vs. 15.28%, p ≤ 0.001). Low uCl^−^ was a significant predictor of one-year mortality (40.4% vs. 16.7%, p < 0.05) and the composite outcome (HR 2.42, 95% CI 1.43–4.08, p < 0.001). In the multivariable analysis, uCl^−^ was independently associated with the risk of one-year mortality (HR 0.92, 95% CI 0.87–0.98, p < 0.05) and the composite outcome (HR 0.95, 95% CI 0.92–0.99, p < 0.05). Our findings suggest that low uCl^−^ is a marker of more advanced heart failure, activation of the renin–angiotensin–aldosterone system and is related to worse one-year outcomes.

## Introduction

Acute heart failure (AHF) is a clinical condition in which the signs and symptoms of heart failure (HF) suddenly appear or worsen, requiring immediate evaluation and treatment^1^. Urine composition analysis has been shown to provide significant clinical information during the decongestion of AHF patients and is recommended as part of care in that population^2^. To date, chloride (Cl^−^) has often taken a back seat in terms of clinical studies concerning HF and fluid dynamics, with sodium (Na^+^) and potassium (K^+^) being the focus of those studies^3–5^. However, recently, this has changed, and increasing attention is being placed upon the role of Cl^−^, "chloride theory" and the potential roles of serum Cl^−^ in kidney function and in fluid distribution within the body^6–10^. Urine Cl^−^ (uCl^−^) is the main electrolyte involved in the regulation of renin secretion in the macula densa. Therefore, it is believed to be an instrument directly responsible for the adjustment of renal response and diuresis^11–14^. Thus, clinical data and studies analyzing the relationship between uCl^−^ and worsening HF outcomes will be of evaluative, therapeutic, and prognostic value. Despite a strong pathophysiological background, data on Cl^−^ (both urine or serum) in an AHF setting are scarce. Therefore, our study looks into the relationship between uCl^−^ in terms of selected clinical and laboratory biomarkers, renal function, and patient outcomes in the AHF population. Finally, we suggest how these findings fit into our emerging understanding of the role of Cl^−^ in HF pathophysiology.

## Methods

### Study population

This was a single-center, observational study that was undertaken in the Centre of Heart Diseases, 4th Military Hospital, Wroclaw, Poland between January 2016 and September 2017. All adult patients (≥ 18 years) hospitalized with AHF as the primary cause of hospitalization, treated with intravenous furosemide at admission, who expressed willingness to participate by signing an informed consent form, were enrolled. Exclusion criteria comprised the following: cardiogenic shock, diagnosis of acute coronary syndrome, severe liver disease, end-stage renal disease requiring renal replacement therapy, and evidence of infection. Patient treatment adhered to the ESC guidelines, as per the recommendations of attending physicians^1,15^. The research study was approved by the local ethics committee and was conducted in accordance with the Declaration of Helsinki.

For the purpose of this analysis, the patient population was divided into two groups based on urine Cl^−^: a low uCl^−^ < 115 mmol/L and a high uCl^−^ ≥ 115 mmol/L group, as determined from the first urine sample collected from the patients. Due to the lack of an available, clinically used cut-off point for uCl^−^, the cut-off was established arbitrarily as a value between the median and Youden index cut-point.

### Study design

Following admission to hospital, detailed information concerning patient demographics (including history of HF), clinical history, comorbidities, previous therapies, and physical findings was collected. Dyspnea was measured using a self-reported 10-point Likert scale at the time of hospital admission, where a score of 0 means ‘absence of dyspnea’ and 10 points indicates ‘the most severe dyspnea/maximal dyspnea’. Clinical assessments, alongside venous blood and urine samples, were collected at baseline and the following subsequent timepoints: day 1, day 2, and discharge. The samples were also centrifuged and frozen at − 70 °C for additional prespecified analysis. A thorough literature review was undertaken, and biomarkers were selected on the basis of their relation to pertinent HF and hypochloremic pathophysiological pathways. The selected biomarkers are those involved in inflammation (CRP, IL-6, IL-22, WBC), liver and kidney function (AST, ALT, bilirubin, albumin, creatinine, blood urea), perfusion and congestion (NT-proBNP, lactate), iron status (Fe, total iron binding capacity, sTfR, Ferritin) and cardiac remodeling (follistatin, lipocalin, GDF-15, Galectin-3), gasometry (Na^+^, Osmolarity, Bicarbonate, Total Carbon Dioxide, pH), and RAAS markers (Renin, Aldosterone). Urine biomarkers of interest included sodium, potassium, chloride, urea, and creatinine.

In our study, baseline urine samples were obtained prior to the first in-hospital dose of intravenous furosemide. The only exception was when the patient was unable to provide a sample before furosemide administration, in which case the sample was collected at the first available moment. Samples were subsequently taken at day 1, day 2, and at discharge.

In accordance with the standard clinical practice at the hospital, all patients were instructed to limit their fluid intake to 1.5–2 L over a 24-h period alongside being advised to limit their daily sodium intake during their hospitalization.

The properties of each marker and evidence in HF and hypochloremia have been previously described elsewhere^16–22^. Information regarding the biomarkers of interest was not available to the treating physicians, and neither clinical nor therapeutic decisions were influenced by these results.

### Laboratory measurements in peripheral blood and urine

Laboratory parameters were assessed using standard methods in our laboratory, including plasma NT-proBNP (N-Terminal Pro-B-Type Natriuretic Peptide) (method: immunoenzymatic, Siemens, Marburg, Germany) and troponin (TNI) (method: immunoenzymatic, single Dimension RxLMax, Siemens). Serum sTfR (mg/L) was measured from plasma samples frozen at − 70 °C using immunonephelometry (Siemens Healthcare Diagnostics, Inc., Deerfield, IL, USA). Both renin and aldosterone were assessed at day 1 and at discharge from frozen samples. The upper limit of normal (ULN) for renin was 46 (µIU/mL) (method: chemiluminescent immunoassay-CLIS, LIASON), and for aldosterone, it was 23.2 (ng/dL) (method: chemiluminescent immunoassay-CLIS, LIASON). The remaining biomarkers of interest were quantified using the Quantikine ELISA Immunoassay kit by R&D Systems. This assay employs the quantitative sandwich enzyme immunoassay technique to assess the following proteins: GDF-15 (also known as macrophage inhibitory cytokine-1 (MIC-1)) (n = 79), Follistatin (FS), Lipocalin-2 (NGAL) (n = 159), Il-6, and Il-22 (n = 159). The measurement of absorbance was performed using the Synergy/HTX multi-mode reader analyzer. The research was carried out in the laboratory of the Department of Clinical Pharmacology at Wroclaw Medical University.

### Study outcomes

The clinical endpoints of the study were:

(i) In-hospital mortality
(ii) One-year mortality
(iii) Composite endpoint of one-year mortality and rehospitalization for heart failure

### Clinical follow-up

Discharged patients were followed-up at the HF clinic for at least one year. Information regarding rehospitalizations and survival status was obtained directly from patients or their relatives (telephone contact), from the HF clinic database, from the hospital system or from the national citizen registry by the investigators, who were blinded to the biomarker results. There were no instances of patient loss during the follow-up period.

### Statistical analysis

Continuous variables with a normal distribution were presented using means and ± standard deviation, variables with skewed distribution were described by medians with [upper and lower quartiles], and categorized variables were given as numbers and percentages. The statistical significance of differences between groups was assessed using the t-test, Mann–Whitney U-test, or Kruskal–Wallis test. The normality of the distributions for these variables was evaluated by three different statistical tests: the Kolmogorov–Smirnov test, Lillefors test, and the Shapiro–Wilk test. To determine the suggested cutoff value of uCl^−^ in our population, we performed an Area Under Curve (AUC) analysis and determined the Youden index cut-point. Variance Inflation Factor was calculated to establish the degree of collinearity between analyzed variables (uNa^+^ and uCl^−^). Cox proportional hazards models were used to calculate the hazard ratios (HR) with their corresponding 95% confidence intervals (95% CI) for all-cause mortality. Multivariable analyses were adjusted for age, ejection fraction, systolic blood pressure at admission, hemoglobin, NT-proBNP, and blood urea nitrogen. The impact of uCl^−^ on the study outcomes (one-year mortality and composite outcome) was analyzed both as a continuous variable and as a categorical variable. Kaplan–Meier survival curves were constructed to demonstrate survival probability. The p < 0.05 level was considered statistically significant. Statistical analyses were performed using STATISTICA 12 (StatSoft Polska Sp. Z o.o., Krakow, Poland).

### Institutional review board statement

The research study was approved by the ethics committee of Wroclaw Medical University (387/2015) and was conducted in accordance with the Declaration of Helsinki.

### Informed consent

Informed consent was obtained from all subjects involved in the study.

## Results

### Baseline characteristics

The study population consisted of 248 patients, of which 182 (73.4%) were male, with a mean (± SD) age of 70.1 ± 12.6 years. The mean left ventricle ejection fraction (LVEF) was 37 ± 14%, and ischemic HF etiology was present in 124 (50%) patients. On admission, the mean systolic blood pressure, serum Na^+^, hemoglobin, and serum creatinine were 134 ± 31 mmHg, 139 ± 4 mmol/L, 13.3 ± 2.0 g/dL, and 1.36 ± 0.52 mg/dL, respectively. The median [upper and lower quartiles] plasma concentrations of NT-proBNP and troponin I were 5618 (3431–11,750) pg/mL and 0.06 (0.03–0.16) ng/mL, respectively. Baseline laboratory tests and concomitant medications are presented in Table 1. The mean uCl^−^ level at admission was 130.7 ± 65.6 mmol/L, with low uCl^−^ observed in 104 (41.9%) patients. The cutoff to define the low uCl^−^ group was set at < 115 mmol/L. The suggested Youden cut-point was 113 mmol/L (refer to Supplementary Fig. 1).

**Table 1:** Baseline characteristics of the population.

Parameter	Population	Low uCl^−^(n = 104)	High uCl^−^(n = 144)	p
Sex (male)	182 (73.4%)	104 (72.2%)	78 (75.0%)	0.625
Age (years)	70.2 ± 12.6	69.3 ± 12.6	70.6 ± 12.7	0.432
De *novo* AHF	104 (41.9%)	31 (29.8%)	70 (50.7%)	0.001
Coronary artery disease	124 (50%)	60 (59.4%)	64 (48.5%)	0.100
Hypertension	198 (79.8%)	78 (77.2%)	120 (85.1%)	0.117
Length of hospitalization (days)	8 ± 6	10 ± 8	7 ± 4	< 0.001
Dyspnea at admission (points)	8.1 ± 2.2	8.1 ± 2.2	8.0 ± 2.2	0.986
Ischemic etiology of heart failure	124 (50%)	60 (59.4%)	64 (48.5%)	0.097
Heart rate (beat/minute)	90 ± 24	90.1 ± 23.7	90.7 ± 24.6	0.867
LVEF (%)	37 ± 14	34.8 ± 14.4	38.8 ± 13.1	0.055
SBP at admission (mmHg)	134 ± 31	128.6 ± 28.8	138.1 ± 32.9	0.020
DBP at admission (mmHg)	79 ± 16	75.8 ± 15.8	81.4 ± 16	0.005
SBP at 24 h (mmHg)	122 ± 23	119 ± 22	124 ± 23	0.115
Hemoglobin (g/dL)	13.3 ± 2.0	13.3 ± 2.1	13.3 ± 2.2	0.966
WBC (G/L)	9.2 ± 4.5	9.4 ± 5.4	9.1 ± 3.6	0.554
PLT (G/L)	210 ± 88	204 ± 77	214 ± 95	0.377
AST (IU/L)	28 (22–41)	29 (22–41)	27 (22–41)	0.647
ALT (IU/L)	31 (21–56)	30 (22–56)	31 (22–58)	0.973
Bilirubin (mg/dL)	1.4 ± 1.1	1.4 ± 1.0	1.3 ± 1.1	0.259
Serum Na^+^ (mmol/L)	139 ± 4	138 ± 4.5	140 ± 4	< 0.001
Serum K^+^ (mmol/L)	4.24 ± 0.7	1.47 ± 0.6	4.2 ± 0.6	0.960
Serum Cl^−^ (mmol/L)	111.6 ± 5.5	110.2 ± 6.0	112.9 ± 4.7	0.003
Blood Urea (mg/dL)	59.7 ± 31.3	68 ± 36	53 ± 25	< 0.001
C-reactive protein (mg/L)	7.7 (4.0–18.9)	7.8 (2.9–17.4)	7.5 (4.8–20)	0.218
NT-proBNP(pg/mL)	5618 (3431–11,750)	5258 (2958–9980)	6283 (4074–12,496)	0.033
Troponin I (ng/mL)	0.06 (0.03–0.16)	0.06 (0.02–0.14)	0.06 (0.03–0.19)	0.320
Creatinine at 24 h (mg/dL)	1.31 ± 0.49	1.4 ± 0.6	1.2 ± 0.4	0.006
Intravenous furosemide at admission	248 (100%)	104 (100%)	144 (100%)	
Furosemide median dose at admission [q1–q3]*	60 (40–80)	60 (40–100)	40(40–80)	0.21
Beta blockers (yes)**	150 (60%)	59 (57%)	91 (63%)	0.305
RASi (yes)**	157 (63%)	62 (60%)	95 (66%)	0.306
MRA (yes)**	60 (24%)	19 (18%)	41 (29%)	0.061

*SBP* systolic blood pressure, *DBP* diastolic blood pressure, *LVEF* left ventricular ejection fraction, *WBC* white blood cell count, *PLT* platelet count, *AST* aspartate transferase, *ALT* alanine transaminase.

*Or other loop diuretic equivalent.

**Prior to hospitalization.

### Comparison of clinical and basic laboratory characteristics by uCl^−^

Lower levels of systolic and diastolic blood pressure on admission were observed in the low uCl^−^ group: 128.6 ± 28.8 vs 138.1 ± 32.9 mmHg, p = 0.02 and 75.8 ± 15.8 vs. 81.4 ± 16 mmHg, p < 0.005, respectively. There were no statistically relevant differences in heart rate (HR) or reported dyspnea on admission between both groups. However, patients with low uCl^−^ presented significantly lower serum Na^+^ and lower serum Cl^−^ when compared with the high uCl^−^ group with 138 ± 4.5 vs. 140 ± 4 mmol/L, p ≤ 0.001 and 110.2 ± 6.0 vs 112.9 ± 4.8 mmol/L, p = 0.003, respectively. The analysis of kidney function tests revealed statistically significant differences between the low uCl^−^ and high uCl^−^ groups, and so, in terms of creatinine at admission, creatinine at 24 h after admission and blood urea at admission, these values were significantly higher in the low uCl^−^ group with 1.47 ± 0.6 vs. 1.28 ± 0.4 mg/dL, p = 0.005; 1.4 ± 0.6 vs. 1.2 ± 0.4 mg/dL, p = 0.006 and 68 ± 36 vs. 53 ± 25 mg/dL, p ≤ 0.001, respectively. No significant differences in liver function tests, blood count and troponin, all analyzed on admission, could be observed between both groups. However, albumin was lower in the low uCl^−^ group, with values of 3.7 ± 0.4 vs. 3.8 ± 0.4 (g/dL), p = 0.031. There was a statistically significant difference in the length of hospitalization between both groups, with 10 ± 8 in the low uCl^−^ group vs. 7 ± 4 days, p = 0.001 (Table 1).

### Comparison of selected biomarkers between uCl^−^ profiles

No significant difference was observed in selected biomarkers related to inflammation, including WBC, CRP, IL-6, and IL-12, between the low uCl^−^ and high uCl^−^groups (Table 2). Of the several remodeling markers examined, lipocalin/NGAL and follistatin differed significantly between the low uCl^−^ and high uCl^−^ groups. Lipocalin/NGAL levels were higher in the low uCl^−^ group (93.6 ± 54.8 vs. 76.3 ± 51.6 ng/mL, p = 0.04), while follistatin levels were lower in the high uCl^−^ group (2102.7 ± 1391.8 vs. 2714.9 ± 1815.5 pg/mL, p = 0.005). NT-proBNP was significantly lower on admission in the low uCl^−^ group, with values of 5258 (2958–9980) vs 6283 (4074–12,496) pg/mL, p = 0.033. However, the reverse was observable at discharge with higher NT-proBNP levels in the low uCl^−^ group (3545 (2143–7750) vs 2903 (1694–5211) pg/mL, p = 0.024).

**Table 2:** Comparison of selected biomarkers between low uCl^−^ group versus high uCl^−^ group.

Variable	Low uCl^−^(n = 104)	High uCl^−^(n = 144)	p
Infection/inflammation			
C-reactive protein (mg/L)	7.8 (2.9–17.4)	7.5 (4.8–20)	0.218
IL-6 (pg/mL)	24.1 ± 43.2	18.4 ± 34.0	0.787
IL-22 (pg/mL)	14.8 ± 23.0	16.2 ± 39.3	0.253
WBC (G/L)	9.4 ± 5.4	9.1 ± 3.6	0.554
Liver and kidney function tests			
AST at admission (IU/L)	29.0 (22–41)	27 (21–41)	0.647
ALT at admission (IU/L)	30 (22–56)	31 (21–58)	0.973
Total bilirubin at admission (mg/dL)	1.4 ± 1.0	1.3 ± 1.1	0.259
Albumin (mg/dL)	3.7 ± 0.4	3.8 ± 0.4	0.031
Serum creatinine at admission (mg/dL)	1.47 ± 0.6	1.28 ± 0.4	0.005
Blood Urea (mg/L	68 ± 36	53 ± 25	< 0.001
Remodeling, congestion and other markers			
Lactate at admission (mmol/L)	2.3 ± 1.5	2.1 ± 0.8	0.275
NT-proBNP at admission (pg/mL)	5258 (2958–9980)	6283 (4074–12,496)	0.033
NT-proBNP at discharge (pg/mL)	3545 (2143–7750)	2903 (1694–5211)	0.024
Lipocalin (NGAL)	93.6 ± 54.8	76.3 ± 51.6	0.04
Follistatin (pg/mL)	2102.7 ± 1391.8	2714.9 ± 1815.5	0.005
GDF-15 (pg/mL)	4614.5 ± 1552.9	4672.3 ± 1611.5	0.874
Galectin-3 (ng/mL)	30.0 ± 42.2	26.7 ± 24.5	0.443
Iron status			
Fe (µg/dL)	52.4 ± 25.0	57.8 ± 32.1	0.186
Total iron binding capacity (µg/dL)	347.7 ± 62.0	346.9 ± 72.7	0.935
sTfR at admission (mg/L)	2.2 ± 0.9	1.8 ± 0.7	< 0.001
Ferritin (µg/L)	160.5 ± 139.0	187.8 ± 155.3	0.191
Gasometry			
pH	7.5 ± 0.1	7.4 ± 0.1	0.408
Serum osmolarity	277.4 ± 11.6	282.8 ± 9.1	< 0.001
Bicarbonate (mmol/L)	23.7 ± 3.5	24.4 ± 2.8	0.082
Renin–Angiotensin–Aldosterone system			
Renin at day-1 (µIU/mL)	70.0 (9.1–300.2)	17.6 (4.4–61.9)	0.001
Renin at discharge (µIU/mL)	47.6 (13.0–343.6)	44.7 (10.9–152.2)	0.159
Aldosterone at day-1 (ng/dL)	13.1 (7.9–27.4)	9.4 (6.2–13.5)	< 0.001
Aldosterone at discharge (ng/dL)	11.3 (6.6–18.4)	8.8 (5.8–13.8)	< 0.001

*IL-6* interleukin-16, *IL-22* interleukin-22, *WBC* white blood cell count, *AST* aspartate transferase, *ALT* alanine transaminase, *NT-proBNP* N-terminal pro B-type natriuretic peptide, *GDF-15* growth/differentiation factor 15, *sTfR* soluble transferrin receptor.

Regarding iron status, only sTfR at admission displayed a statistically relevant difference between the two groups, with higher levels recorded in the low uCl^−^ group (2.2 ± 0.9 vs. 1.8 ± 0.7 (mg/L), p ≤ 0.001).

### The renin–angiotensin–aldosterone system in different uCl^−^ profiles

The renin–angiotensin–aldosterone system (RAAS) demonstrated significant differences in the majority of variables analyzed. Renin levels at day 1, but not at discharge, were higher in the low uCl^−^ group (70.0 (9.1–300.2) vs 17.6 (4.4–61.9) µIU/mL, p = 0.001 and 47.6 (13.0–343.6) vs 44.7 (10.9–152.2) µIU/mL, p = 0.159, respectively). Aldosterone levels at day 1 and discharge were significantly higher in the low uCl^−^ group (13.1 (7.9–27.4) vs 9.4 (6.2–13.5) and 11.3 (6.6–18.4) vs 8.8 (5.8–13.8) ng/dL), respectively, both p < 0.001 (Table 2).

In the multivariable analysis (corrected for systolic and diastolic blood pressure at admission, renin, serum Na^+^, serum osmolarity, serum creatinine, and NT-proBNP), only aldosterone assessed at admission to the hospital was found to be significantly associated with urine chloride (beta- 0.22), p = 0.018.

### Comparison of urine biomarkers between the low uCl^−^ and high uCl^−^ groups

We observed in the low uCl^−^ group, the following lower biomarker levels: uNa^+^; 67.3 ± 28.1 vs. 100.6 ± 33.6 mmol/L, p ≤ 0.001, uK^+^; 26.9 ± 13.4 vs. 33.1 ± 16.2 mmol/L, p = 0.002, urine cations (Na^+^  + K^+^); 88.9 ± 21 vs 161 ± 70 mmol/L, p < 0.001 and uCl^−^/urine cations index; 0.96 ± 0.19 vs 1.33 ± 0.99, p < 0.001 (Table 3). The correlation between uCl^−^ and uNa^+^ in our population is high r ~ 0.7, p < 0.001, however the variance inflation factor in the model was low, ranging between 1.02 and 1.03.

**Table 3:** Comparison of selected urine biomarkers between the low uCl^−^ and high uCl^−^ groups.

Variable	Low uCl^−^ (n = 104)	High uCl^−^ (n = 144)	p
Urine Na (mmol/L)	67.6 ± 28.0	100.5 ± 33.7	< 0.0001
Urine K (mmol/L)	26.9 ± 13.4	33.0 ± 16.2	0.002
Urine cations (Na^+^ + K^+^) (mmol/L)	88.9 ± 21	161.0 ± 70	< 0.001
Urine urea (mg/dL)	897.6 ± 611.6	971.3 ± 709.5	0.403
Urine creatinine (mg/dL)	73.7 ± 65.6	82.9 ± 82.3	0.356
Urine Cl^−^/urine cations index	0.96 ± 0.19	1.33 ± 0.99	< 0.001

### Differences in in-hospital outcomes between the low uCl^−^ and high uCl^−^ groups

**Figure 1:**
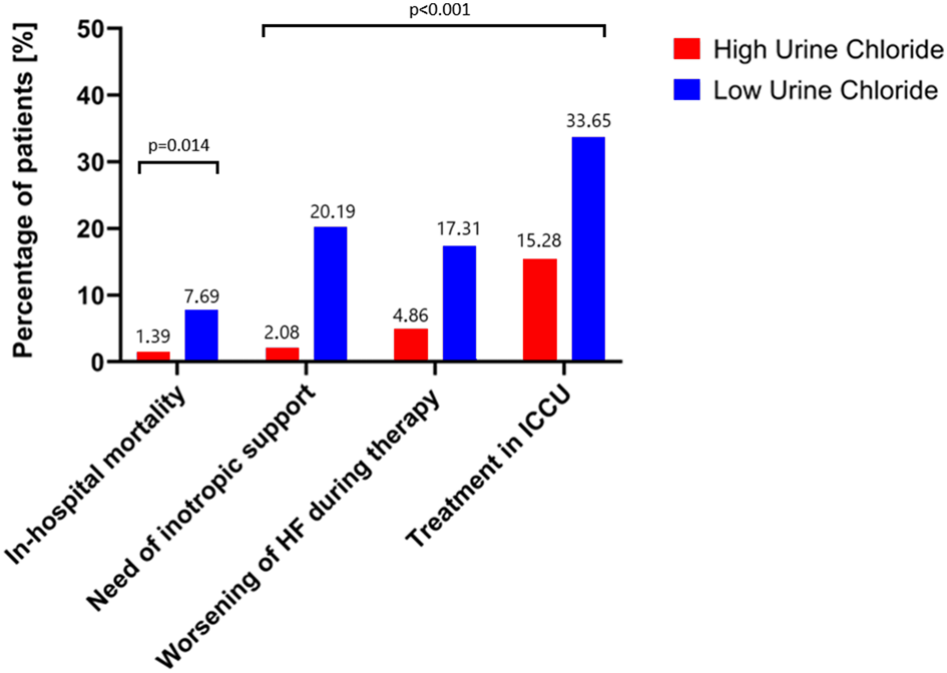
In-hospital outcomes in low (red) vs. high (blue) uCl^−^ groups.

All in-hospital outcomes were significantly worse in the low uCl^−^ group. In terms of in-hospital mortality, eight (7.7%) patients experienced the event in the low uCl^−^ group vs. two (1.4%) in the high uCl^−^ group, p = 0.014. Other clinical outcomes were also worse in the low uCl^−^ group, such as the need for inotropic support (20.19% low uCl^−^ vs. 2.08% high uCl^−^, p ≤ 0.001), worsening of HF during therapy (17.31% low uCl^−^ vs. 4.86% high uCl^−^, p ≤ 0.001), and the need for treatment in an intensive cardiac care unit (33.65% low uCl^−^ vs. 15.28% high uCl^−^ group, p ≤ 0.001) (Fig. 1).

### The prognostic significance of low uCl^−^ in AHF

**Figure 2:**
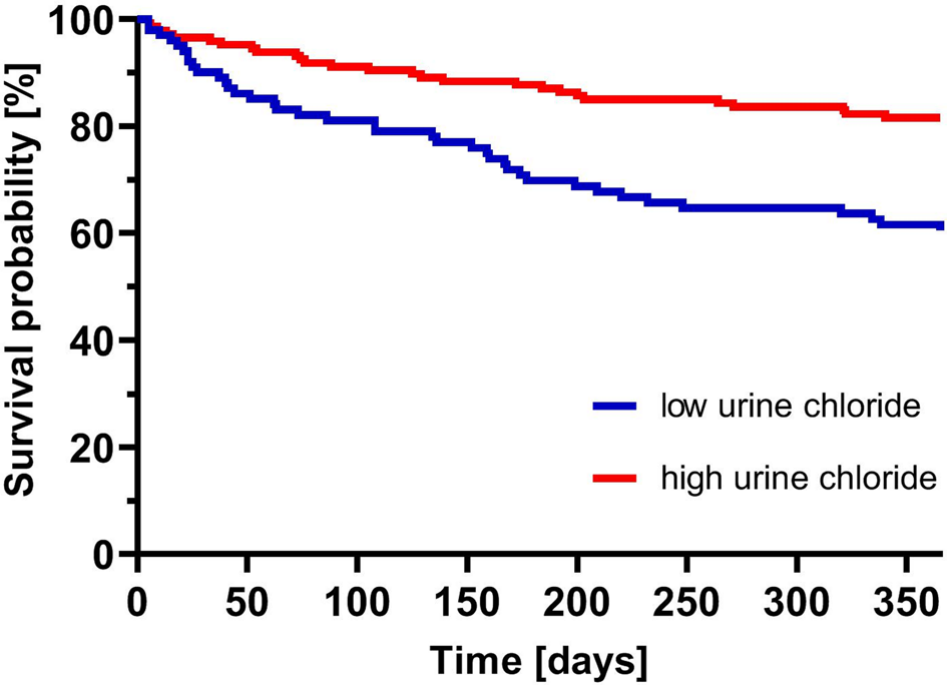
Probability of survival over time. Probability of survival over time (days) for patients in both the low uCl^−^ group (blue) and the high uCl^−^ group (red).

**Figure 3:**
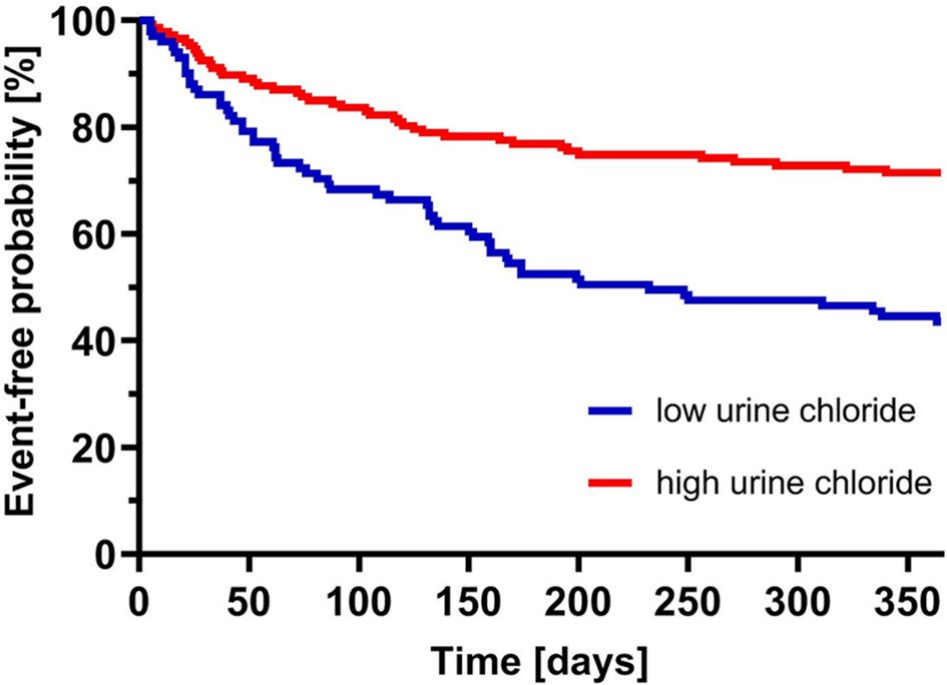
Probability of survival without any event over time. Probability of survival without any adverse events over time (days) for patients in the low uCl^−^ group (blue) and the high uCl^−^ group (red).

One-year mortality in the low uCl^−^ group compared to the high uCl^−^ group was 40.4% (42 events) vs. 16.7% (24 events), p < 0.05. The probability of survival in both groups is presented in Fig. 2, and the probability of survival without any event in both groups is presented in Fig. 3. The low uCl^−^ group was at significantly higher risk of one-year mortality and the composite outcome, with a hazard ratio (HR) of 95% confidence interval (95% CI) 2.42 (1.43–4.08) and 2.20 (1.44–3.36), respectively, both p < 0.001 (Table 4). In the multivariable model (after adjustments for systolic blood pressure, serum creatinine, ejection fraction, age, bilirubin, and NT-proBNP at admission) uCl^−^ , evaluated as a continuous variable (per 10 mmol/L change) was related to the risk of both one-year mortality and the composite outcome, with HRs of (95% CI) 0.92 (0.87–0.98) and 0.95 (0.92–0.99), respectively, both p < 0.05 and when evaluated per standard deviation, comparable, although weaker, results were observed with HRs of (95%CI) 0.56 (0.38–0.84) and 0.74 (0.57–0.98), respectively, both p < 0.05 (Table 4). Comparable results were observed when the multivariable model adjusted for gender, estimated glomerular filtration rate (eGFR) at admission, atrial fibrillation at admission, NT-proBNP at admission, chronic obstructive pulmonary disease (COPD) and troponin I (TNI) at admission (Supplementary Table A).

**Table 4:** The impact of urine chloride on study outcomes.

	χ^2^	Multivariable model*	
		HR (95% CI)	P
One year mortality			
Urine Cl^−^ (per 10 mmol/L)	32.9	0.92 (0.87–0.98)	0.008
Urine Cl^−^ (per Standard Deviation)	34.4	0.56 (0.38–0.84)	0.004
Low urine Cl^−^ (yes vs no)	35.0	2.42 (1.43–4.08)	< 0.001
One year mortality or heart failure hospitalization (whichever occurred first)			
Urine Cl^−^ (per 10 mmol/L)	37.0	0.95 (0.92–0.99)	0.047
Urine Cl^−^ (per standard deviation)	37.5	0.74 (0.57–0.98)	0.036
Low urine Cl^−^ (yes vs no)	45.9	2.20 (1.44–3.36)	< 0.001

*Multivariable model adjusted for systolic blood pressure, serum creatinine, ejection fraction, age, bilirubin, NT-proBNP at admission.

In a subgroup of patients (n = 172) that underwent simultaneous assessment of Cl^−^ in urine and serum we examined the impact of these markers on prognosis. In a multivariable model that included uCl^−^, serum Cl^−^ as well as other variables (SBP, serum creatinine, ejection fraction, age and NT-proBNP at admission), only serum creatinine and uCl^−^ were independent prognostic markers, the HR (95% CI) for uCl^−^ in the model was 0.99 (0.98–0.99), p = 0.008 (see Supplementary Table B).

In a bivariable model that included uNa^+^ and uCl^−^, both variables were independent prognostic markers in our population (see Supplementary Table C). Moreover, both markers were independent prognosticators of one-year mortality, but uNa^+^ outperformed uCl^−^ in terms of identifying patients reaching the composite endpoint (see Supplementary Tables D−G).

Importantly, uCl^−^ was significantly related to outcomes even when analyzed as per 10 mmol/L change, per standard deviation change and as a continuous variable (see Supplementary Material).

## Discussion

The primary outcome of our study is the adverse association of low uCl^−^ levels with various clinical outcomes when compared to the high uCl^−^ group. In patients with AHF, the current standard for renal function monitoring includes serum creatinine analysis, glomerular filtration rate (eGFR) estimation, and urine excretion volumes^23,24^. Recently, there has been a growing interest in incorporating urine composition, particularly spot uNa^+^, into the early assessment algorithm for AHF patients^1–25^. Our study aligns with previous reports that highlight the prognostic potential of uNa^+^ in predicting response to diuretics, one-year mortality, and rehospitalizations, thereby establishing the significant diagnostic value of uCl^−^. This diagnostic value is further supported by our observation that uNa^+^ and uCl^−^ both function as independent prognostic markers.

While uNa^+^ has received substantial attention as an indicator of resistance to diuretic treatment and worsening HF symptoms leading to increased mortality^28–30^, uCl^−^ has been overlooked as a valuable biomarker, despite its significant role in HF pathophysiology^31^. Most previous studies regarding Cl^−^ have primarily focused on serum concentration^7^, and to the best of our knowledge, our study is among the few to explore the relationship between uCl^−^ and patient outcomes^32,33^. Our analysis of urine biomarkers reveals a positive correlation between all analyzed electrolytes, consistent with trends observed in serum levels. Significantly, after multivariable analysis of uCl^−^ and serum Cl^−^ only uCl^−^ remained an indicator of prognostic value.

Recent emerging studies^6,7,34^ have introduced the “chloride theory,” which posits that changes in serum chloride concentration are the primary determinant of alterations in plasma volume and the RAAS in worsening HF and its therapeutic resolution. Our observations regarding uCl^−^ correspond with this emerging theory, particularly through our identification of low uCl^−^ being associated with increased RAAS activation and fluid balance dysfunction, as evidenced by statistically significant decreases in serum Na^+^ and osmolarity in the low uCl^−^ group. Notably, the most prominent differentiation between the low uCl^−^ and high uCl^−^ groups pertains to the RAAS, showing marked differences in relevant parameters. Cl^−^ plays a pivotal role in the RAAS within the kidney and is known to exhibit an inverse relationship with renin^35–37^. The persistent activation of the RAAS in HF is linked to adverse long-term effects, such as ventricular remodeling, water and salt retention, and higher mortality^38–43^. Since the drafting of this manuscript, a further study regarding ‘chloride theory’ has been published. In the study, which comprised a cohort of 29 patients with acute HF, uCl^−^ concentration demonstrated an inverse correlation with RAAS activity^44^. Additionally, the patients involved in the study were then divided into two groups based on the difference between serum and uCl^−^ concentrations, namely an excretion group (low renal Cl^−^ avidity, higher uCl^−^) and an absorption group (high renal Cl^−^ avidity, lower uCl^−^). The absorption group was associated with impaired renal function, increased cardiac burden, and neurohormonal activity, thereby further substantiating the importance of Cl^−^ in HF pathophysiology.

Similarly, in our study, patients with low uCl^−^ concentration exhibited several characteristics indicative of a higher HF burden or more advanced disease stage, including RAAS activation, lower blood pressure, hyponatremia, and elevated NT-proBNP levels at discharge. Additionally, the low uCl^−^ group demonstrated worse renal function, as evidenced by higher creatinine, blood urea, and lipocalin/NGAL values. These findings are consistent with the well-established relationship between kidney function and heart failure, although kidney health during the HF treatment phase remains somewhat ambiguous^45^.

HF triggers a detrimental cycle of cardiac burden, neurohormonal and inflammatory responses, and progressive multiorgan dysfunction leading to mortality^38,46–51^. Low uCl^−^ levels appear to be indicative of an organism under distress and, within the context of HF, may serve as a prognostic indicator for unfavorable outcomes. In fact, patients with a low uCl^−^ concentration exhibited several characteristics indicative of a higher HF burden or more advanced disease stage and in-hospital indices of poor prognosis. This underscores the importance of monitoring uCl^−^ levels in HF patients and suggests its potential utility as a valuable biomarker for predicting outcomes in this patient population.

Nevertheless, it is essential to acknowledge the limitations of our study. It was a single-center post hoc analysis constrained by a limited sample size, drawn solely from the Polish resident population. The study was designed to optimally assess the first in-hospital urine sample before any initial intravenous dose of furosemide, which occurred in the majority of cases. However, there were instances where certain patients received diuretics while in transit in the ambulance (n = 12, 5%), or the first available urine sample was obtained upon admission to the ward (after receiving furosemide). Since the initial urine samples exhibited relatively high levels of uNa^+^ and uCl^−^, it is important to consider that various confounding factors may have already affected chloride excretion during the pre-hospital phase. This could possibly be attributed to an increased dosage of oral loop diuretics routinely taken by many patients once their congestion status started to deteriorate. Furthermore, the analyzed biomarkers may introduce selection bias, and as is common in comparable studies, male patients were overrepresented. In our paper the cut-off value to distinguish between high and low uCl^−^ was chosen arbitrarily, but this cut-off has almost the same specificity and sensitivity to the cut-off of 113 mmol/L calculated from the ROC analysis. A longitudinal assessment (trajectory) of urine chloride during hospitalization could provide further insights into its role in AHF pathophysiology. Additionally, confirmation of the differentiation between the low uCl^−^ and high uCl^−^ groups necessitates multicenter studies and a more extensive patient cohort.

## Conclusion

Chloride has transitioned from being an overlooked “cousin” of sodium (Na^+^) to becoming one of the more intriguing markers for assessing acute heart failure (AHF) and predicting prognosis. Our study has shown that low urine Cl^−^ excretion during an AHF episode serves as an indicator of more advanced HF and activation of the RAAS and is related to worse one-year outcomes. A simple urine Cl^−^ test, therefore, could be used to identify those patients who are in need of more intensive and specific treatment.

### Supplementary Information

Supplementary Information.

## Data Availability

All relevant data are contained within the manuscript.
