# Supplementary material for: The role of urine chloride in acute heart failure
Source: Sci Rep. 2024 Jun 18;14:14100. doi: 10.1038/s41598-024-64747-5 (PMC11189561; doi:10.1038/s41598-024-64747-5)

**The role of urine chloride in acute heart failure.**

Sylwia Nawrocka-Millward^1*^, Jan Biegus^1,2^, Marat Fudim^2-4^, Guzik Mateusz^1,2^, Gracjan Iwanek^1,2^, Piotr Ponikowski^1,2^, Robert Zymliński^1,2^

1. University Clinical Hospital, Wroclaw, Poland
2. Institute of Heart Diseases, Wroclaw Medical University, Poland
3. Division of Cardiology, Duke University Medical Center, Durham, NC, USA
4. Duke Clinical Research Institute, Durham, NC, USA

* Correspondence: smnawrocka@gmail.com; Tel.: +48 509 943 930

**Supplementary Material**

**Supplementary Table A.** The impact of urine chloride on study outcomes (Expanded Table).

|  | **ꭓ^2^** | **Multivariable model*** | |
| --- | --- | --- | --- |
| **One year mortality** | | | |
|  |  | **HR (95% CI)** | **P** |
| Urine Cl^-^ (per 10 mmol/l) | 30.2 | 0.92 (0.86-0.98) | 0.009 |
| Urine Cl^-^ (per Standard Deviation) | 29.7 | 0.53 (0.34-0.82) | 0.005 |
| Low urine Cl^-^ (yes vs no) | 36.0 | 2.82 (1.65-4.28) | 0.001 |
| **One year mortality or heart failure hospitalization** (whichever occurred first) | | | |
| Urine Cl^-^ (per 10 mmol/l) | 24.6 | 0.96 (0.91-0.99) | <0.05 |
| Urine Cl^-^ (per Standard Deviation) | 19.3 | 0.73 (0.54-0.99) | 0.041 |
| Low urine Cl^-^ (yes vs no) | 37.2 | 2.45 (1.60-3.75) | <0.0001 |

* Multivariable model adjusted for gender, estimated glomerular filtration rate at admission, atrial fibrillation at admission, NT-proBNP at admission, chronic obstructive pulmonary disease and troponin I at admission.

**Supplementary Table B.** The impact of uCl^-^  and Serum Creatinine in the multivariable model on study outcomes.

|  | **ꭓ^2^** | **Multivariable model*** | |
| --- | --- | --- | --- |
| **One year mortality** | | | |
|  |  | **HR (95% CI)** | **P** |
| uCl^-^ (mmol/l) | 33.0 | 0.99 (0.98-1.0) | 0.008 |
| Serum Creatinine (mg/dl) | 33.0 | 1.56 (1.08-2.27) | 0.02 |

* Multivariable model adjusted for gender, estimated glomerular filtration rate at admission, atrial fibrillation at admission, NT-proBNP at admission, chronic obstructive pulmonary disease and troponin I at admission.

**Supplementary Table C.**  Impact of uCl^-^ and uNa^+^ on one-year mortality in a bivariable model.

|  | **ꭓ^2^** | **Bivariable model** | |
| --- | --- | --- | --- |
| **One year mortality** | | | |
|  |  | **HR (95% CI)** | **P** |
| uCl^-^ (mmol/l) | 21.4 | 0.99 (0.98-1.0) | 0.009 |
| uNa^+^ (mmol/l) | 21.4 | 0.99 (0.98-1.0) | 0.002 |

**Supplementary Table D.** The impact uCl^-^ and uNa^+^ on study outcomes per 10 mmol/l (Expanded Table).

|  | **ꭓ^2^** | **Multivariable model*** | |
| --- | --- | --- | --- |
| **One year mortality** | | | |
|  |  | **HR (95% CI)** | **P** |
| Urine Cl^-^ (per 10 mmol/l) | 42.0 | 0.94 (0.88-0.99) | 0.029 |
| Urine Na^+^ (per 10 mmol/l) | 42.0 | 0.87 (0.81-0.95) | <0.001 |
| **One year mortality or heart failure hospitalization** (whichever occurred first) | | | |
| Urine Cl^-^ (per 10 mmol/l) | 38.9 | 0.97 (0.96-1.00) | 0.136 |
| Urine Na^+^ (per 10 mmol/l) | 38.9 | 0.89 (0.84-0.95) | <0.001 |

* Multivariable model adjusted for gender, estimated glomerular filtration rate at admission, atrial fibrillation at admission, NT-proBNP at admission, chronic obstructive pulmonary disease and troponin I at admission.

**Supplementary Table E.** The impact uCl^-^ and uNa^+^ on study outcomes per Standard Deviation (Expanded Table).

|  | **ꭓ^2^** | **Multivariable model*** | |
| --- | --- | --- | --- |
| **One year mortality** | | | |
|  |  | **HR (95% CI)** | **P** |
| Urine Cl^-^ (per Standard Deviation) | 41.6 | 0.63 (0.42-0.94) | 0.024 |
| Urine Na^+^ (per Standard Deviation) | 41.6 | 0.63 (0.48-0.83) | <0.001 |
| **One year mortality or heart failure hospitalization** (whichever occurred first) | | | |
| Urine Cl^-^ (per Standard Deviation) | 34.5 | 0.82 (0.62-1.08) | 0.163 |
| Urine Na^+^ (per Standard Deviation) | 34.5 | 0.65 (0.52-0.81) | <0.001 |

* Multivariable model adjusted for gender, estimated glomerular filtration rate at admission, atrial fibrillation at admission, NT-proBNP at admission, chronic obstructive pulmonary disease and troponin I at admission.

**Supplementary Table F.** The impact uCl^-^ and uNa^+^ on study outcomes per 10 mmol/l.

|  | **ꭓ^2^** | **Multivariable model*** | |
| --- | --- | --- | --- |
| **One year mortality** | | | |
|  |  | **HR (95% CI)** | **P** |
| Urine Cl^-^ (per 10 mmol/l) | 41.2 | 0.94 (0.89-0.99) | 0.027 |
| Urine Na^+^ (per 10 mmol/l) | 41.2 | 0.90 (0.83-0.97) | 0.005 |
| **One year mortality or heart failure hospitalization** (whichever occurred first) | | | |
| Urine Cl^-^ (per 10 mmol/l) | 46.8 | 0.97 (0.93-1.01) | 0.154 |
| Urine Na^+^ (per 10 mmol/l) | 46.8 | 0.91 (0.85-0.97) | 0.002 |

* Multivariable model adjusted for systolic blood pressure, serum creatinine, ejection fraction, age, bilirubin, NT-proBNP at admission.

**Supplementary Table G.** The impact uCl^-^ and uNa^+^ on study outcomes (per Standard Deviation).

|  | **ꭓ^2^** | **Multivariable model*** | |
| --- | --- | --- | --- |
| **One year mortality** | | | |
|  |  | **HR (95% CI)** | **P** |
| Urine Cl^-^ (per Standard Deviation) | 42.4 | 0.64 (0.44-0.92) | 0.017 |
| Urine Na^+^ (per Standard Deviation) | 42.4 | 0.68 (0.52-0.89) | 0.005 |
| **One year mortality or heart failure hospitalization** (whichever occurred first) | | | |
| Urine Cl^-^ (per Standard Deviation) | 47.6 | 0.81 (0.62-1.06) | 0.124 |
| Urine Na^+^ (per Standard Deviation) | 47.6 | 0.71 (0.57-0.88) | 0.002 |

* Multivariable model adjusted for systolic blood pressure, serum creatinine, ejection fraction, age, bilirubin, NT-proBNP at admission

**Supplementary Table H.** The impact of urine chloride grouped by the median uCl^-^ at admission on the outcomes.

|  | **ꭓ^2^** | **Multivariable model*** | |
| --- | --- | --- | --- |
| **One year mortality** | | | |
|  |  | **HR (95% CI)** | **P** |
| Low urine Cl^-^ (yes vs no) | 31.8 | 2.12 (1.25-3.60) | 0.005 |
| **One year mortality or heart failure hospitalization** (whichever occurred first) | | | |
| Low urine Cl^-^ (yes vs no) | 43.0 | 2.02 (1.32-3.08) | 0.001 |

* Multivariable model adjusted for systolic blood pressure, serum creatinine, ejection fraction, age, bilirubin, NT-proBNP at admission.

**Figure 1.** The Area Under Curve (AUC) and Youden cut-off point in the population.


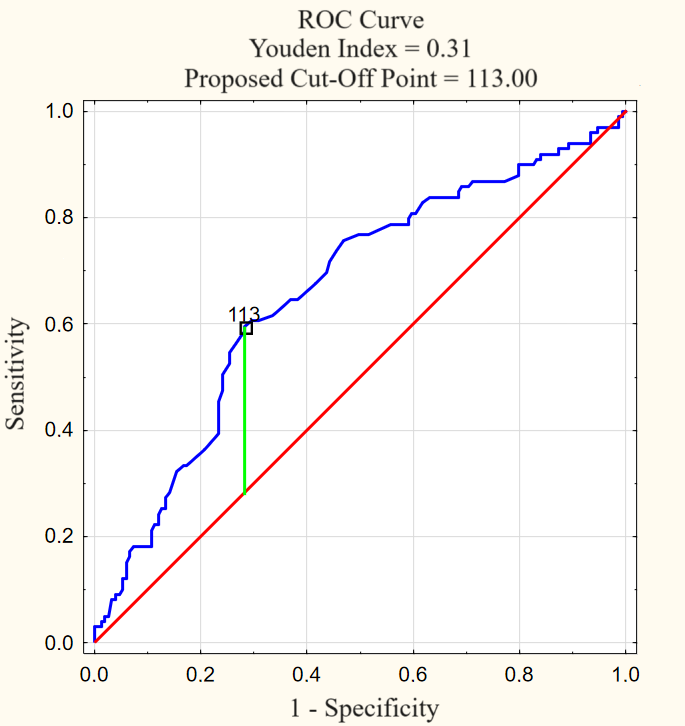

Supplement: Supplementary file 1 — Supplementary Information. [file 41598_2024_64747_MOESM1_ESM.docx]
